# Evaluation of Multimodal Algorithms for the Segmentation of Multiparametric MRI Prostate Images

**DOI:** 10.1155/2020/8861035

**Published:** 2020-10-20

**Authors:** Ying-Hwey Nai, Bernice W. Teo, Nadya L. Tan, Koby Yi Wei Chua, Chun Kit Wong, Sophie O'Doherty, Mary C. Stephenson, Josh Schaefferkoetter, Yee Liang Thian, Edmund Chiong, Anthonin Reilhac

**Affiliations:** ^1^Clinical Imaging Research Centre, Yong Loo Lin School of Medicine, National University of Singapore, Singapore; ^2^Nanyang Junior College, Singapore; ^3^St. Joseph's Institution International, Singapore; ^4^Anglo-Chinese Independent, Singapore; ^5^Joint Department of Medical Imaging, University Health Network, Toronto, Canada; ^6^Siemens Medical Solutions USA, Inc., Molecular Imaging, Knoxville, TN, USA; ^7^Department of Diagnostic Imaging, National University Hospital, Singapore; ^8^Department of Surgery (Urology), Yong Loo Lin School of Medicine, National University of Singapore, Singapore; ^9^Department of Urology, National University Hospital, Singapore

## Abstract

Prostate segmentation in multiparametric magnetic resonance imaging (mpMRI) can help to support prostate cancer diagnosis and therapy treatment. However, manual segmentation of the prostate is subjective and time-consuming. Many deep learning monomodal networks have been developed for automatic whole prostate segmentation from T2-weighted MR images. We aimed to investigate the added value of multimodal networks in segmenting the prostate into the peripheral zone (PZ) and central gland (CG). We optimized and evaluated monomodal DenseVNet, multimodal ScaleNet, and monomodal and multimodal HighRes3DNet, which yielded dice score coefficients (DSC) of 0.875, 0.848, 0.858, and 0.890 in WG, respectively. Multimodal HighRes3DNet and ScaleNet yielded higher DSC with statistical differences in PZ and CG only compared to monomodal DenseVNet, indicating that multimodal networks added value by generating better segmentation between PZ and CG regions but did not improve the WG segmentation. No significant difference was observed in the apex and base of WG segmentation between monomodal and multimodal networks, indicating that the segmentations at the apex and base were more affected by the general network architecture. The number of training data was also varied for DenseVNet and HighRes3DNet, from 20 to 120 in steps of 20. DenseVNet was able to yield DSC of higher than 0.65 even for special cases, such as TURP or abnormal prostate, whereas HighRes3DNet's performance fluctuated with no trend despite being the best network overall. Multimodal networks did not add value in segmenting special cases but generally reduced variations in segmentation compared to the same matched monomodal network.

## 1. Introduction

Prostate cancer (PCa) is the most frequently diagnosed cancer with the second highest mortality in men worldwide in 2018 [1]. Commonly employed PCa screening methods such as the prostate-specific antigen test are subjective and inaccurate, leading to unnecessary invasive prostate biopsy or misdiagnosis of patients with aggressive PCa [2, 25]. Multiparametric magnetic resonance imaging (mpMRI) is noninvasive and together with the Prostate Imaging-Reporting and Data System (PI-RADS) assessment guidelines (PI-RADS v2) allows for better diagnosis, localization, risk stratification, and staging of PCa [3, 4].

Image segmentation can help to localize prostate boundaries for radiotherapy, monitor disease progression by measuring the prostate volume, support multimodal registration, identify the region of interest for computer-aided detection (CAD), or support the staging of PCa using PI-RADS [5, 6]. However, accurate segmentation is difficult as the prostate anatomic structure is highly varied and complex [7], especially the transition zone (TZ), which has a multitude of structural variations among subjects [8]. Moreover, manual delineation of the prostate boundaries is tedious and time-consuming and is subjected to inter- and intraobserver variations [5].

Automatic segmentation using machine learning (ML) or deep learning (DL) is faster than manual segmentation and can localize the prostate more consistently, objectively, and efficiently. However, automatic segmentation may yield poor results when image quality is suboptimal due to motion, intensity inhomogeneity, partial volume effects, and poor tissue contrast [9]. Insufficient training data, high data variability [10], or presence of implants also yields poor outcomes. Despite its limitations, automatic segmentation speeds up the segmentation process and improves the diagnostic workflow for radiologists. Moreover, the loss of accuracy using semiautomatic methods was shown to be below the measured interobserver variability in manual segmentation of the whole prostate gland (WG) from T2-weighted (T2w) MR images [11].

Many DL and ML algorithms have been developed for medical segmentation and were submitted to various “Grand Challenges in Medical Imaging,” such as the PROMISE12 challenge [5]. Some of these DL networks have been extensively evaluated to understand the impact of the network architectures on the accuracy of prostate segmentation [5, 6]. However, these networks are monomodal, using only T2w images as input to delineate WG only. The use of multimodal networks can substantially increase the segmentation accuracy as shown recently in brain tumors [12]. For PI-RADS grading, multiparametric MR images are acquired and graded independently in the peripheral zone (PZ) and TZ before obtaining a single PI-RADS score [3]. As such, the automatic segmentation of the WG into PZ and the central gland (CG) may further facilitate PI-RADS assessment and CAD performance for PCa detection.

Our goal is to develop a CAD system to support automatic PI-RADS grading using multiparametric MR images, by first segmenting the prostate into PZ and CG to facilitate parameter weighting for lesion detection and PI-RADS grading. In this work, we aimed to identify suitable networks for segmenting the prostate by comparing monomodal and multimodal DL networks. The networks evaluated were DenseVNet (monomodal), HighRes3DNet (monomodal and multimodal), and ScaleNet (multimodal). The added value of multimodal networks was thoroughly evaluated in segmenting the WG into various subregions—PZ and CG and apex, middle, and base of the WG. Particularly, the segmentation of special cases, such as subjects who underwent transurethral resection of the prostate (TURP), was investigated. For TURP cases, part of CG and sometimes part of the PZ are removed, resulting in a larger urine channel. The mask typically consists of a hole within the mask and may consist of only PZ. The amount of data required to optimally train a DL network depends on the complexity of the problem, the learning algorithm, and the amount of variation in data. Although networks are typically exposed to as many different cases as possible, the networks were also evaluated with varying numbers of subject data for network development to determine if multimodal networks also add value in reducing the number of training data required.

## 2. Materials and Methods

### 2.1. Comparison of DL Networks

DenseVNet is a monomodal network consisting of convolutional units with key characteristics such as batch-wise spatial dropout, dense feature stacks, V-network upsampling and downsampling, dilated convolutions, and an explicit spatial prior [14]. The network was first implemented for multiorgan segmentation on abdominal CT images and was reported to yield significantly higher dice similarity coefficient (DSC) from 0.63 to 0.96 for all organs than VNet, VoxResNet, and DEEDS+JLF [14]. HighRes3DNet primarily uses dilated convolutions and residual connections to create an end-to-end mapping from image volume to voxel-level dense segmentation [15]. It was initially proposed for the parcellation of brain structures and achieved DSC of 0.84 ± 0.02. ScaleNet is a multimodal network and comprises backend and frontend, where the backend is made up of HighRes3DNet, while the frontend merges the data from the backend to the frontend independently of the number of input modalities. The network was compared with the BraTS'13 winners' challenge results and achieved the highest DSC of 0.88 for glioma segmentation using various MR images [12]. The three networks are available on NiftyNet (Version 0.5.0) [13], a TensorFlow-based convolutional neural network (CNN) platform. NiftyNet implements a patch sampling strategy to extract the necessary information for better convergence and higher performance generalization.

### 2.2. Image Data

Among the publicly available datasets (refer to Supplementary Table 1), we focused on datasets with large subject data and were acquired with the closest imaging protocol recommendations of PI-RADS version 2 [3], where DWI was acquired with three *b* values to model the ADC maps. As such, we randomly selected 160 subjects' MR data, without any preferences, from the PROSTATEx Challenge dataset (https://prostatex.grand-challenge.org/). 77% of the subjects had lesions, of which 66% had lesions graded as PI‐RADS < 3, with lesion information provided from the PROSTATEx Challenge dataset. Transverse morphological T2w, apparent diffusion coefficient (ADC), and diffusion-weighted imaging (DWI) images were selected as inputs as these are the most important sequences in mpMRI for PI-RADS evaluation. The images were acquired on the Siemens 3T MRI scanners (either MAGNETOM Trio or Skyra) without an endorectal coil. T2w images had a voxel size of approximately 0.5 × 0.5 × 3.6 mm^3^. DWI images were acquired with a single-shot echoplanar imaging sequence with a voxel size of 2 × 2 × 3.6 mm^3^, with diffusion encoding gradients in three directions. The DWI images were acquired using *b* values of 50, 400, and 800 s/mm^2^, from which ADC maps were calculated by the scanner software.

We subsequently corrected the T2w images for nonuniformity and resliced the ADC and DWI images to the T2w image space using SPM12 (https://www.fil.ion.ucl.ac.uk/spm/). The image intensities of all images were linearly scaled to within 0-1000 only as further image intensity normalization and whitening can be implemented within NiftyNet before network training. The field of view was then cropped within a fixed position and resliced to obtain a final matrix size of 192 × 192 × 46 with a voxel size of 0.5 × 0.5 × 2 mm^3^, covering the entire prostate for all subjects to reduce the computational burden and limit the amount of unwanted background voxels. All images were checked to ensure that there were no missing prostate regions in the T2w, ADC, and DWI images after cropping.

Prostate masks were manually drawn by two students (80 each) trained in segmenting the prostate into CG and WG on the T2w images using the Medical Imaging Interaction Toolkit (MITK) software (https://www.mitk.org). The PZ mask was then obtained by subtracting the CG from the WG to remove discrepancies at the PZ-CG boundary. All masks were subsequently corrected by a research fellow with 2 years of experience in segmenting prostates. Our ground truth masks have been assessed slice by slice and verified by an experienced medical physicist with over 10 years' experience and deemed sufficiently accurate. Out of 160 subject data, there were only two cases with TURP and one with prostatic utricle cyst (PUC). We thus identified these subjects and a subject with an abnormally large prostate and inhomogeneous image intensity as special cases. The dataset was split into 120, 20, and 20 for training, validation, and test. For the training data, the WG, PZ, and CG volumes are 53.3 ± 26.7 [15.9, 152.8] cm^3^, 18.4 ± 7.6 [6.6, 47.4] cm^3^, and 34.9 ± 23.6 [6.3, 118.2] cm^3^. The test data consisted of WG, PZ, and CG volumes of 64.1 ± 45.3 [21.2, 199.2] cm^3^, 18.9 ± 8.3 [5.4, 37.6] cm^3^, and 45.1 ± 42.4 [5.5, 174.2] cm^3^. The training data consisted of one subject with TURP but no subject with PUC. The test data includes a different subject with TURP and one subject with PUC, and 76% of the test subjects have one lesion.

For monomodal networks, only T2w images were used as input, while for multimodal networks, DWI images with a *b* value of 800 s/mm^2^, ADC images or both, were included in addition to the T2w images. DWI with a *b* value of 800 s/mm^2^ was selected as it was the only *b* value that fitted the ESUR 2012 guidelines and higher *b* value shows greater tissue contrast [16]. The number of subjects for training was varied from 20 to 120 in steps of 20 for DenseVNet and HighRes3DNet, with the forced inclusion of the same subject with TURP each time. ScaleNet was not evaluated due to technical difficulties in running this network with this scenario. The validation and test data remained unchanged.

### 2.3. Data Analysis

Three commonly applied segmentation metrics, namely, DSC, absolute relative volume difference (aRVD), and average Hausdorff distance (AHD), were used to compare the automatically segmented masks and manually drawn masks [5, 6]. All evaluation metrics were calculated for PZ, CG, and WG individually for the 20 test subjects and the apex, middle, and base of the WG measured within 0-15^th^, 16-84^th^, and 85-100^th^ percentiles of all slices of the ground truth masks, respectively. The evaluation was carried out using an in-house program written in python.

DSC and aRVD were calculated using the volumes of the automatically segmented masks (*X*) and the manually drawn ground truth masks (*Y*):

(1) $$\begin{array}{c} \text{DSC}\left(X,Y\right)=\frac{2\left|X\cap Y\right|}{\left|X\right|+\left|Y\right|}, \end{array}$$

(2) $$\begin{array}{c} \text{aRVD}\left(X,Y\right)=\left|\frac{X-Y}{Y}\right|. \end{array}$$

AHD measures the boundary mismatch between the segmented mask (*X*) and the ground truth (*Y*):

(3) $$\begin{array}{c} \text{AHD}=\max\left(D\left(X,Y\right),D\left(Y,X\right)\right). \end{array}$$

*D*(*X*, *Y*) denotes the directed Hausdorff distance from the boundary in *X* to the closest boundary in *Y*. The Hausdorff distance was averaged over all the points to make AHD less sensitive to outliers. AHD was determined in 2D and averaged within specified slices using a program in python [17].

Statistical analysis was carried out using Student's paired *t*-tests with two-tailed distribution across the different configurations in MATLAB (MathWorks, Inc., US, Version R2017b), with significance defined as *p* ≤ 0.05.

### 2.4. Network Training and Optimization

The networks were run on CPU (Dell OptiPlex 9020) and were kept unchanged for ease of comparison with other works. The three networks were each optimized with about 80 different hyperparameter configurations (https://niftynet.readthedocs.io/en/dev/config_spec.html), within the computational feasibility of the CPU, with the optimal hyperparameter configuration shown in Table 1. During optimization, the networks were evaluated using the DSC calculated for the WG of the 20 test data.

## 3. Results

### 3.1. Monomodal vs. Multimodal Networks

**Figure 1:**
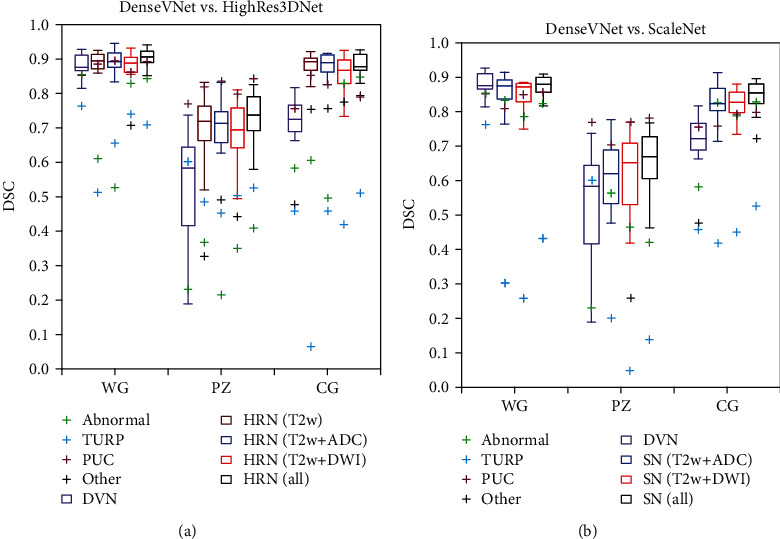
DSC of all the segmentation of the WG, PZ, and CG regions of 20 subjects, generated using the optimized network of (a) DenseVNet (DVN) against HighRes3DNet (HRN) and (b) DVN against ScaleNet (SN) with different image input combinations.

Figure 1 shows the DSC distribution for DenseVNet with HighRes3DNet and ScaleNet using different input combinations. The special cases, abnormal prostate, TURP, and PUC, were plotted separately from the others as highlighted with green, blue, and magenta crosses. Overall, HighRes3DNet yielded the highest DSC across WG, PZ, and CG regions, with smaller variability, followed by DenseVNet and ScaleNet. ScaleNet segmented the subject with TURP poorly, which appeared as the only one outlier in WG. HighRes3DNet segmented the subject with TURP and abnormal prostate poorly, though the DSC of these cases was higher than that of ScaleNet. DenseVNet showed lower DSC in PZ and CG but achieved the highest DSC for WG of the TURP case. All network configurations were able to segment the WG, PZ, and CG of the subject with PUC well. The use of T2w, ADC, and DWI as inputs yielded the highest DSC with smaller variability for ScaleNet and fewer outliers for HighRes3DNet. Thus, HighRes3DNet and ScaleNet, with all images as inputs, were selected as the optimal input configurations for subsequent evaluations unless stated otherwise. No significant difference was found within each region across all configurations of HighRes3DNet and ScaleNet with *p* ≤ 0.05.

### 3.2. Segmentation of PZ, CG, and WG

HighRes3DNet performed the best, achieving the highest DSC and lowest aRVD and AHD for WG, PZ, and CG (Table 2). ScaleNet was the second best for PZ and CG segmentation, while DenseVNet was the second best for WG segmentation. Although ScaleNet performed slightly poorer than DenseVNet for WG, DenseVNet performed much poorer for PZ and CG with comparably higher aRVD and AHD and lower DSC. This indicated that multimodal networks are more capable of segmenting the prostate subregions, especially at the PZ-CG border, with additional information provided by the ADC and DWI images.

### 3.3. Segmentation of the Apex, Middle, and Base of WG

All three networks performed the best in the middle region and the worst in the base region of WG (Table 3). In our study, we used 0-15th and 85-100th percentile for apex and base because of the wide variation in prostate volumes in our dataset, which may account for the lower DSC in apex and base. HighRes3DNet performed the best from the apex to the base in general with the highest DSC and lowest AHD, but DenseVNet yielded the lowest aRVD in the base. DenseVNet yielded smaller variations, followed by HighRes3DNet and then ScaleNet.

### 3.4. Statistical Differences in Segmentation

Statistical analysis was carried out within WG, PZ, and CG regions and within the apex, middle, and base of the WG region as shown in Table 4. No significant difference was found in the WG across all three networks but was found in DSC and aRVD between monomodal DenseVNet and multimodal HighRes3DNet and ScaleNet in PZ and CG for all subjects. Removing the three special cases, significant differences were observed between HighRes3DNet and DenseVNet, as well as between HighRes3DNet and ScaleNet. Significant difference in AHD was found only between HighRes3DNet against ScaleNet in the apex and middle and between DenseVNet and HighRes3DNet in the PZ for all cases and in the PZ region with HighRes3DNet against DenseVNet and ScaleNet.

### 3.5. Segmentation of Special Cases

**Figure 2:**
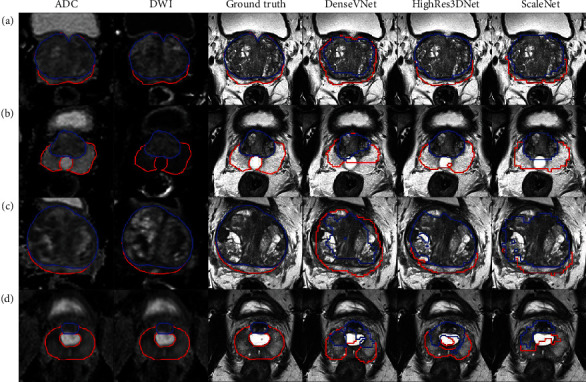
Transverse views of the ADC and DWI images with the respective segmentations of PZ (red) and CG (blue) of ground truth, DenseVNet, HighRes3DNet, and ScaleNet for 4 subjects with (a) the highest WG's DSC across all networks, (b) TURP, (c) abnormally large prostate volume with uneven image intensities, and (d) PUC on T2-weighted images.

Figure 2 shows the slices with the best PZ and CG segmentations generated using DenseVNet, HighRes3DNet, and ScaleNet for test subjects with the highest DSC in WG (Figure 2(a)), TURP (Figure 2(b)), large prostate volume with uneven intensity (Figure 2(c)), and PUC (Figure 2(d)). HighRes3DNet yielded good segmentation for all 4 subjects and managed to segment close to the borders of the WG and between CG and PZ. However, it included the urinary tract for the subject with TURP, which appeared as an outlier in Figure 1(a). It also misclassified parts of the CG in the subject with tissue heterogeneity and included the PUC in PZ segmentation. ScaleNet generated crude segmentation but segmented reasonably well for the subjects with abnormally large prostate volume and PUC but could not segment the subject with TURP, which appeared as an outlier in Figure 1(b). ScaleNet yielded better PZ and CG boundaries but poorer WG segmentation than DenseVNet. The segmentation outputs of the remaining 16 test subjects are shown in Supplementary Figure 1.

### 3.6. Impact of the Number of Training Datasets

**Figure 3:**
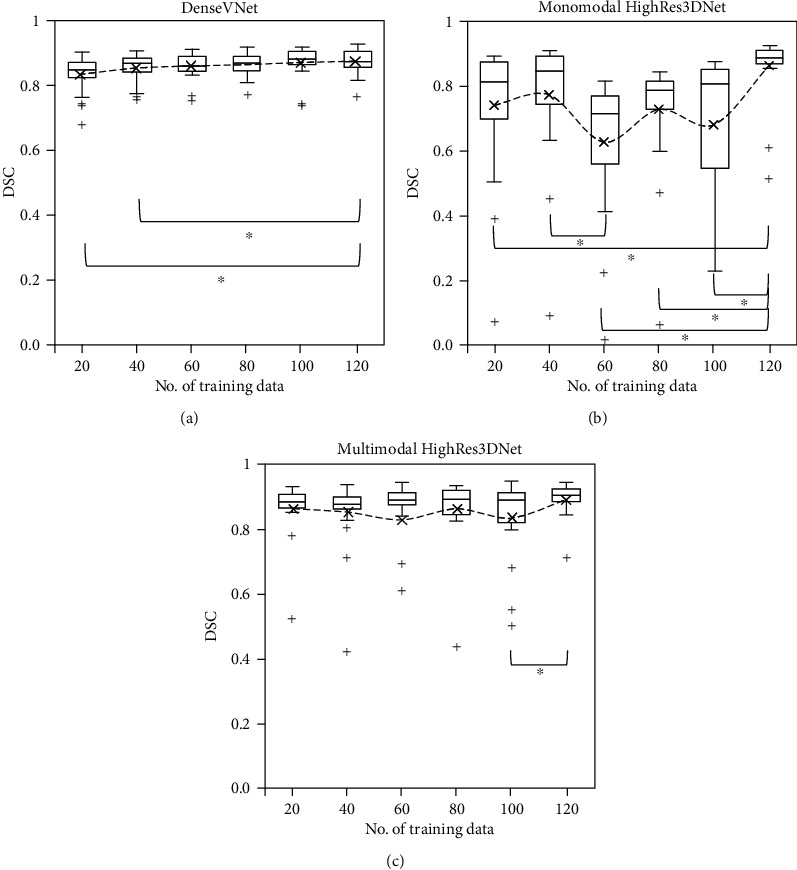
DSC of WG segmentations of 20 subjects generated using the optimized network of (a) DenseVNet, (b) monomodal HighRes3DNet, and (c) multimodal HighRes3DNet with 20 to 120 training data, in steps of 20. The dotted lines show the mean, while the line within the box shows the median value. ^∗^Significant difference was observed between the 2 specified datasets with *p* < 0.05.

Figure 3 shows the DSC distribution of 20 test subjects as a function of the number of training data input into DenseVNet, monomodal HighRes3DNet, and multimodal HighRes3DNet. Generally, the performance of DenseVNet improved slightly with increasing number of training data with significant differences between 20 and 40 against 120 training data only. However, the improvement plateaued after 100 training data. No obvious trend could be observed with monomodal or multimodal HighRes3DNet. Significant differences were observed between monomodal HighRes3DNet networks trained with different numbers of data, particularly with 120 training data. HighRes3DNet performed poorly with 100 training data but performed very well with 120 training data with generally higher DSC, fewer outliers, thus yielding a significant difference between 100 and 120 training data. Multimodal HighRes3DNet outperformed monomodal HighRes3DNet with higher DSC for special cases, with much smaller variation in segmentation for normal cases even with a small number of training data of 20.

## 4. Discussion

To our knowledge, this is the first report wherein monomodal and multimodal CNNs are directly compared. The results from previously published studies are reported in Table 5. The list is not exhaustive and mostly includes results obtained with images scanned without an endorectal coil.

Ghavami et al. compared the accuracy of the prostate segmentation of six CNNs: UNet, VNet, HighRes3DNet, HolisticNet, DenseVNet, and Adapted UNet [6]. Their HighRes3DNet and DenseVNet networks were trained on a total of 173 T2w images with 15,000 iterations, yielding DSC of 0.89 and 0.88 for the WG of 59 test subjects (Table 5). We trained these networks with 120 subject data with 1000 and 2000 iterations and obtained comparable DSC of 0.875 and 0.890 for the WG of 20 subjects with DenseVNet and multimodal HighRes3DNet (Table 2). The performance of DenseVNet plateaued after 100 training data (Figure 3(a)); thus, the DSC obtained was similar though their networks were trained with more data and iterations. Our monomodal HighRes3DNet yielded a lower DSC of 0.858 than that obtained by Ghavami et al. [6], but our multimodal HighRes3DNet yielded the same DSC of 0.890, indicating that multimodal inputs improve the segmentation but the overall performance is dependent on the network architecture. Moreover, statistical differences were only found between monomodal DenseVNet and multimodal HighRes3DNet and ScaleNet in PZ and CG (Table 4), indicating that multimodal networks added value by generating better PZ and CG segmentations but did not improve the WG segmentation.

Most reported DL networks were monomodal, with T2w images as input and yielded DSC ranging from 0.73 to 0.93 (Table 5). 3D Multistream UNet uses three T2w images acquired in the axial, coronal, and sagittal planes to segment the PZ and CG [24]. The network is relatively similar to our multimodal network. It, however, yielded slightly higher DSC in WG and PZ of 0.905 and 0.799 for the Siemens data. Our multimodal HighRes3DNet attained a lower DSC of 0.890 and 0.712 for WG and PZ for 20 subjects (Table 2). This slightly higher performance may be attributed to the larger number of training data used (297 vs. 120) or the larger data variation in our dataset or the nature of the input images. Cascaded 2D UNet first generated a rough segmentation using DWI images with *k*-means clustering, before using 2D-UNet to segment the T2w image to obtain the WG mask, which was then used as input into another 2D-UNet to segment the PZ only. Their network was trained using 76 images and 100 iterations and obtained DSC of 0.927 and 0.793 for the WG and PZ of 51 subject data (Table 5). Similarly, Cheng et al. [23] used a 2-step segmentation trained with 100 T2w images, first using the active appearance model to get an approximation, followed by a five-layered CNN to refine the segmentation and achieved a high DSC of 0.925 averaged over 20 unseen test data (Table 5).

Khan et al. [25] applied class-weighting approach to reduce class imbalance, thus yielding slightly higher DSC for classical UNet and SegNet, though they trained their networks with different number of subjects (Table 5). Dense-2 UNet [26] produced similar performance as the cascaded 2D UNet [18]. Note that the cascaded UNet used by Aldoj et al. [26] for comparison with Dense-2 UNet differed from that used by Zhu et al. [18] in that a rough segmentation was not generated for input into the cascaded network, which may account for the lower DSC. The architecture of Dense-2 UNet included a transition layer after each dense block, and the input into the block was the concatenated output from all the layers within the previous block, which helps in compressing the information while retaining information that may be lost due to convolutional operations. This may indicate that focusing the network using a rough mask to learn from “more useful” information enabled the network to learn more efficiently by reducing background or unwanted tissues or by retaining important network information. Despite the additional information from multimodal inputs, multimodal networks still included a significant amount of background or unwanted tissues.

We yielded lower DSC in the apex and base compared to that reported in the PROMISE12 challenge despite comparable WG DSC [5]. The DSC, aRVD, and AHD of the apex and base of WG segmentation from multimodal HighRes3DNet and ScaleNet were generally not significantly different from those of monomodal DenseVNet (Table 4). This indicated that apex and base segmentations were more affected by the general network architecture and multimodality inputs may not improve apex and base segmentations. Although ADC and DWI images have different lesion contrast, both multimodal HighRes3DNet and ScaleNet included all the lesions within the appropriate regions. Moreover, multimodal HighRes3DNet segmented close to the boundary with good PZ and CG differentiation; it could not segment subjects with PUC and TURP well. No significant difference was observed visually in segmenting the prostate with and without lesions across the networks with the average DSC of 0.86 vs. 0.89 (*p* value = 0.49).

Prostate segmentation was most difficult for cases with TURP, followed by abnormal prostate volume with uneven image intensity, even though all training data included one subject with TURP. Prostates with generally larger volume or with uneven image intensities were poorly segmented by the network sometimes (highlighted with black crosses in Figure 1). However, no general trend could be observed. The subject with the largest prostate volume and with uneven image intensity within the prostate region only was selected as a special case as it was poorly segmented on all networks except for a few combinations. Although multimodal HighRes3DNet segmented the prostates of subjects with PUC and abnormal prostate volume reasonably well, it did not manage to segment the subject with TURP better than DenseVNet with DSC of 0.709 vs. 0.764. Special attention is required to validate the automatic segmentation of these cases. Increasing data specific to these special cases may improve these segmentations.

DenseVNet was able to yield reasonably high DSC even when trained with 20 subject data, including subjects with TURP, abnormal prostate volume, and PUC with DSC > 0.65 (Figure 3(a)). However, both multimodal and monomodal HighRes3DNet could not segment the subject with TURP with 60 training data. Higher DSC with smaller variation was achieved with multimodal HighRes3DNet compared to monomodal HighRes3DNet regardless of the number of training data. This showed that multimodal networks reduced the variation in segmentations but the overall performance and number of training data required are dependent on the network architecture. Multimodal HighRes3DNet can yield highly accurate segmentation close to the prostate boundary with good PZ and CG segmentation for regular cases, with higher DSC and smaller AHD (Table 2), but requires a large number of training data for accurate segmentation (Figure 3(c)). Therefore, for general segmentation of the prostate or in cases with limited training data, DenseVNet might be a better network.

## 5. Conclusions

We investigated the added values of multimodal networks, compared to monomodal networks, in segmenting the prostate gland and its two main subregions. Multimodal networks improved the boundary segmentation of the subregions but not the whole gland and not the apex and base of the whole gland compared to monomodal networks. Despite the increase in inputs, the number of training data required for multimodal networks to yield decent segmentation was not reduced, although the variability in DSC of output segmentation was reduced. The use of multiple inputs did not help in segmenting special cases such as TURP and abnormally large prostate volume. However, multimodal networks can yield highly accurate regional segmentation with sufficient training data. Our multimodal networks did not yield higher DSC compared to reported “focused” 2-step network that first generates a rough mask as input into the second network, which enabled the network to learn more efficiently. Our results may be translated to support network development for the automatic segmentation of other biomedical images.

## Figures and Tables

**Table 1:** Optimal hyperparameter configurations for DenseVNet, ScaleNet, and HighRes3DNet.

Hyperparameters	DenseVNet	ScaleNet	HighRes3DNet
Activation function	ReLU	Leaky ReLU	Leaky ReLU
Optimizers	Adam	Nesterov momentum	Adam
Batch size	8	4	4
Interpolation	Linear	B-spline	B-spline
Learning rate	10^−3^	10^−2^	10^−2^
Loss function	Dice	Dice no square	Dice plus cross-entropy
Whitening	True	True	False
Normalization	False	False	True
Regularization type	L1	L2	L2
Sample per volume	1	10	8
Volume padding size	16	24	16
Window sampling	Resize	Resize	Resize
Spatial window size	(56, 56, 56)	(40, 40, 48)	(96, 96, 48)
No. of iterations	2000	1000	1000

**Table 2:** The DSC, aRVD, and AHD (mean ± standard deviation [worst, best]) of the WG, PZ, and CG segmentation for the optimized networks of DenseVNet, HighRes3DNet, and ScaleNet.

Metrics	DenseVNet			HighRes3DNet			ScaleNet		
	WG	PZ	CG	WG	PZ	CG	WG	PZ	CG
DSC	0.875 ± 0.039 [0.764, 0.927]	0.527 ± 0.171 [0.190, 0.769]	0.699 ± 0.096 [0.460, 0.816]	0.890 ± 0.049 [0.709, 0.942]	0.712 ± 0.109 [0.409, 0.843]	0.856 ± 0.090 [0.509, 0.926]	0.848 ± 0.102 [0.433, 0.907]	0.623 ± 0.156 [0.140, 0.782]	0.826 ± 0.082 [0.526, 0.897]
aRVD	0.088 ± 0.055 [0.006, 0.209]	1.119 ± 1.109 [0.052, 4.764]	0.402 ± 0.190 [0.029, 0.933]	0.077 ± 0.086 [0.010, 0.361]	0.188 ± 0.211 [0.011, 0.712]	0.134 ± 0.200 [0.017, 0.921]	0.101 ± 0.139 [0.001, 0.642]	0.263 ± 0.330 [0.009, 1.333]	0.137 ± 0.132 [0.002, 0.541]
AHD	1.901 ± 0.715 [1.009, 3.932]	2.409 ± 1.073 [1.259, 5.470]	2.142 ± 0.968 [1.061, 4.980]	1.792 ± 0.690 [0.994, 3.777]	1.709 ± 0.551 [1.118, 2.866]	1.720 ± 0.622 [0.917, 3.561]	2.084 ± 0.821 [1.092, 4.357]	1.924 ± 0.628 [1.255, 3.284]	1.940 ± 0.767 [1.051, 4.117]

**Table 3:** The DSC, aRVD, and AHD (mean ± standard deviation [worst, best]) of the apex, middle, and base of the WG segmentation for the optimized networks of DenseVNet, HighRes3DNet, and ScaleNet.

Metrics	DenseVNet			HighRes3DNet			ScaleNet		
	Apex	Middle	Base	Apex	Middle	Base	Apex	Middle	Base
DSC	0.757 ± 0.201 [0.000, 0.930]	0.897 ± 0.037 [0.796, 0.946]	0.568 ± 0.295 [0.000, 0.899]	0.788 ± 0.232 [0.050, 0.949]	0.912 ± 0.041 [0.766, 0.957]	0.576 ± 0.281 [0.000, 0.915]	0.694 ± 0.227 [0.042, 0.894]	0.877 ± 0.097 [0.483, 0.937]	0.467 ± 0.303 [0.000, 0.916]
aRVD	0.289 ± 0.228 [0.029, 1.000]	0.094 ± 0.062 [0.003, 0.215]	0.412 ± 0.337 [0.026, 1.000]	0.218 ± 0.270 [0.001, 0.974]	0.076 ± 0.067 [0.002, 0.835]	0.433 ± 0.339 [0.024, 1.000]	0.337 ± 0.269 [0.018, 0.978]	0.086 ± 0.124 [0.001, 0.582]	0.578 ± 0.326 [0.020, 1.000]
AHD	2.775 ± 1.011 [1.414, 4.774]	3.677 ± 0.536 [2.855, 5.102]	3.004 ± 0.970 [1.323, 4.690]	2.521 ± 0.798 [1.500, 4.942]	3.449 ± 0.607 [2.689, 4.848]	2.864 ± 0.967 [1.225, 4.534]	3.086 ± 0.773 [1.658, 4.781]	4.022 ± 0.690 [3.118, 5.565]	3.306 ± 1.157 [1.658, 5.828]

**Table 4:** *p* values generated between two different networks using Student's *t*-test for DSC, aRVD, and AHD for WG, PZ, and CG regions and the apex, middle, and base of the WG segmentations of all subjects and excluding the three special cases in (). ^∗^*p* < 0.05, ^∗∗^*p* < 0.01, and ^∗∗∗^*p* < 0.001.

Metrics	Paired networks	Regions						Within WG regions					
		WG		PZ		CG		Apex		Middle		Base	
DSC	DenseVNet-HighRes3DNet	0.292	(∗)	∗∗∗	(∗∗∗)	∗∗∗	(∗∗∗)	0.657	(0.432)	0.254	(0.060)	0.934	(0.875)
	DenseVNet-ScaleNet	0.274	(0.289)	0.074	(∗∗)	∗∗∗	(∗∗∗)	0.358	(0.680)	0.379	(0.573)	0.291	(0.458)
	HighRes3DNet-ScaleNet	0.104	(∗∗∗)	∗	(∗)	0.280	(∗)	0.204	(0.220)	0.145	(∗)	0.245	(0.355)

aRVD	DenseVNet-HighRes3DNet	0.620	(0.069)	∗∗	(∗∗)	∗∗∗	(∗∗∗)	0.376	(0.203)	0.382	(0.067)	0.847	(0.942)
	DenseVNet-ScaleNet	0.703	(0.209)	∗∗	(∗∗)	∗∗∗	(∗∗∗)	0.544	(0.837)	0.803	(∗)	0.122	(0.237)
	HighRes3DNet-ScaleNet	0.512	(0.610)	0.399	(0.454)	0.955	(0.264)	0.171	(0.140)	0.747	(0.647)	0.176	(0.216)

AHD	DenseVNet-HighRes3DNet	0.627	(0.547)	∗	(0.217)	0.109	(0.075)	0.384	(0.182)	0.217	(0.103)	0.650	(0.709)
	DenseVNet-ScaleNet	0.456	(0.481)	0.089	(0.085)	0.469	(0.477)	0.282	(0.457)	0.085	(0.076)	0.377	(0.421)
	HighRes3DNet-ScaleNet	0.231	(0.209)	0.257	(∗∗)	0.324	(0.232)	∗	(∗)	∗∗	(∗∗)	0.198	(0.265)

**Table 5:** Comparison of previously reported DSC for prostate segmentation using DL networks trained with the stated image inputs and the number of training subject data as reported in the literature. AAM-CNN = active appearance model followed by a CNN. ^#^With an endorectal coil. ^&^With surface coil. ^$^Training and test data are from the same datasets. ^%^Averaged across 2 datasets. *^Β^*Slices instead of subjects. ^*ϵ*^NCI-ISBI 2013 Challenge dataset consisted of PROSTATE-DIAGNOSIS and Prostate-3T datasets (refer to Supplementary Table 1).

Networks	Input images	No. of training iterations	No. of training subjects	No. of test subjects	DSC (WG)	DSC (PZ)	Ref.
UNet	T2w	15,000	173	59	0.84 ± 0.07	—	[6]
VNet					0.88 ± 0.03	—	
HighRes3DNet					0.89 ± 0.03	—	
HolisticNet					0.88 ± 0.12	—	
DenseVNet					0.88 ± 0.03	—	
Adapted UNet					0.87 ± 0.03	—	
ConvNet	T2w	80	141	12	0.862 ± 0.008	—	[8]
Cascaded 2D UNet	DWI (*B* value = 1000 s/mm^2^, preprocessing), T2w	100	76	51	0.927 ± 0.042	0.793 ± 0.104	[18]
DSCNN	T2w		77	4	0.885	—	[19]
PSFCN	T2w	80,000	—	20	0.853 ± 0.032	—	[20]
Volumetric	T2w	10,000	50	30	0.894	—	[21]
ConvNet							
SegNet	T2w	—	19	4	0.73	—	[22]
AAM-CNN	T2w^#^	—	100	20	0.925	—	[23]
3D Multistream	T2w	—	220 (GE)	22	0.882 ± 0.058^$^	0.765 ± 0.115^$^	[24]
UNet	(axial, sagittal, coronal)		330 (Siemens) 550 (Combined)	33 55	0.905 ± 0.027^$^ 0.859 ± 0.075^%^	0.799 ± 0.094^$^ 0.800 ± 0.086^%^	
FCN	T2w^*ϵ*^	—	40 (542*^Β^*)	82*^Β^*	0.866 ± 0.048	0.727 ± 0.051	[25]
SegNet	T2w^&^	—	11 (229*^Β^*)	72*^Β^*	0.843 ± 0.042	0.760 ± 0.039	
UNet					0.884 ± 0.037	0.768 ± 0.033	
DeepLabV3+					0.919 ± 0.020	0.789 ± 0.019	
UNet	T2w	36,952	141	47	0.907 ± 0.07	0.750 ± 0.10	[26]
Cascaded UNet		—			0.871 ± 0.07	0.716 ± 0.10	
PSPNet		—			0.911 ± 0.03	0.771 ± 0.10	
Dense-2 UNet		35,760			0.921 ± 0.03	0.781 ± 0.09	

## Data Availability

The authors do not have permission to share the image data, which was obtained from PROSTATEx Challenge dataset (https://prostatex.grand-challenge.org/). Trained networks and labeled data are available upon request.

## Supplementary Materials

Supplementary Materials Supplementary Table 1: overview of publicly available online prostate-related imaging datasets. Supplementary Figure 1: transverse views of the ADC and DWI images with the respective segmentations of PZ and CG of ground truth, DenseVNet, HighRes3DNet, and ScaleNet for the remaining 16 test subjects.
