# Supplementary material for: Evaluation of Multimodal Algorithms for the Segmentation of Multiparametric MRI Prostate Images
Source: Comput Math Methods Med. 2020 Oct 20;2020:8861035. doi: 10.1155/2020/8861035 (PMC7596462; doi:10.1155/2020/8861035)

**Supplementary Table 1**: Overview of publicly-available online prostate-related imaging datasets. (<https://wiki.cancerimagingarchive.net/display/Public/Wiki>). DWI = Diffusion-weighted imaging, ADC = apparent diffusion coefficient, DCE = dynamic contrast enhanced, PET = positron emission tomography. *Acquired with endorectal coil only. ^#^ Lesion information only. ^$^ Only for some subjects. N/A = Not applicable. U/A = Unavailable.

| Imaging Datasets | PROSTATE-MRI | PROSTATE-DIAGNOSIS | Prostate-3T | PROMISE12 | NaF Prostate | QIN PROSTATE | QIN-PROSTATE-Repeatability | Prostate-MRI-US-Biopsy | Prostate Fused-MRI-Pathology | TCGA-PRAD | SPIE-AAPM-NCI | |
| --- | --- | --- | --- | --- | --- | --- | --- | --- | --- | --- | --- | --- |
|  |  |  |  |  |  |  |  |  |  |  | **PROSTATEx** | **PROSTATEx-2** |
| No of Subjects | 26 | 92 | 64 | 50 | 9 | 22 | 15 | 1151 | 28 | 14 | 346 | 192 |
| Total no of Image Series | 182 | 368 | 64 | 50 | 214 | 319 | 270 | 2799 | 325 | 207 | 18,321 | U/A |
| MR Field-Strength | 3T | 1.5T | 3T | 3T | N/A | 3T | 3T | 3T | 3T | U/A | 3T | 3T |
| Coil-type | Endorectal and phased array surface coil | Endorectal coil | Pelvic phased-array coil |  | N/A | Body array coil and endorectal coil | Transrectal coil | Transabdominal phased array and endorectal coil^$^ | U/A | U/A | Without an endorectal coil | Without an endorectal coil |
| With Contrast Agent | 🗶 | 🗶 | 🗶 | 🗶 | 🗶 | 🗶  (Magnevist) | ✓ | 🗶 | ✓ | 🗶 | ✓ | ✓ |
| With Segmentations | 🗶 | 🗶 | 🗶 | ✓ | 🗶 | 🗶 | ✓ | ✓ | 🗶 | 🗶 | 🗶 | 🗶 |
| With Pathology | 🗶 | 🗶 | 🗶 | 🗶 | 🗶 | 🗶 | 🗶 | ✓ | ✓ | ✓ | ✓^#^ | ✓^#^ |
| Image Types |  |  |  |  |  |  |  |  |  |  |  |  |
| T1-weighted |  | ✓ |  |  |  | ✓ |  |  | ✓ | ✓ |  |  |
| T2-weighted | ✓ | ✓ | ✓ | ✓ |  | ✓ | ✓ | ✓ | ✓ | ✓ | ✓ | ✓ |
| DWI  (B-values s/mm^2^) | ✓  (U/A) |  |  |  |  | ✓*  (500, 1400) | ✓  (0, 1400) | ✓ | ✓  (600) |  | ✓  (50, 400, 800) | ✓  (50, 400, 800) |
| ADC | ✓ |  |  |  |  |  | ✓ |  |  |  | ✓ | ✓ |
| DCE | ✓ | ✓ |  |  |  | ✓ | ✓ | ✓ | ✓ |  | ✓ | ✓ |
| Proton-Density |  |  |  |  |  |  |  |  |  |  | ✓ | ✓ |
| Ultrasound |  |  |  |  |  |  |  | ✓ |  |  |  |  |
| PET/CT | ✓ |  |  |  | ✓ |  |  |  |  |  |  |  |
| References | [1,2] | [1,2] | [1,2] | [3] | [1,2] | [1,2] | [1,2] | [2] | [1,2] | [1,2] | [1,2,5] | [2,5] |

References:

1. Hulsen, Tim. 2019. “An Overview of Publicly Available Patient-Centered Prostate Cancer Datasets.” Translational Andrology and Urology. AME Publishing Company. <https://doi.org/10.1016/j.juro.2018.02.2246>.
2. “Collections - The Cancer Imaging Archive (TCIA) Public Access - Cancer Imaging Archive Wiki.” <https://wiki.cancerimagingarchive.net/display/Public/Collections>.
3. “Details - PROMISE12 - Grand Challenge.” <https://promise12.grand-challenge.org/Details/>.
4. “Overview - PROSTATEx - Grand Challenge.” <https://prostatex.grand-challenge.org/>.
5. “PROSTATEx-2 Challenge.” <https://www.aapm.org/GrandChallenge/PROSTATEx-2/>.

**Supplementary Figure 1**: Transverse views of the ADC, DWI images with the respective segmentations of PZ (red) and CG (blue) of Groundtruth, DenseVNet, HighRes3DNet, and ScaleNet for remaining 16 test subjects.


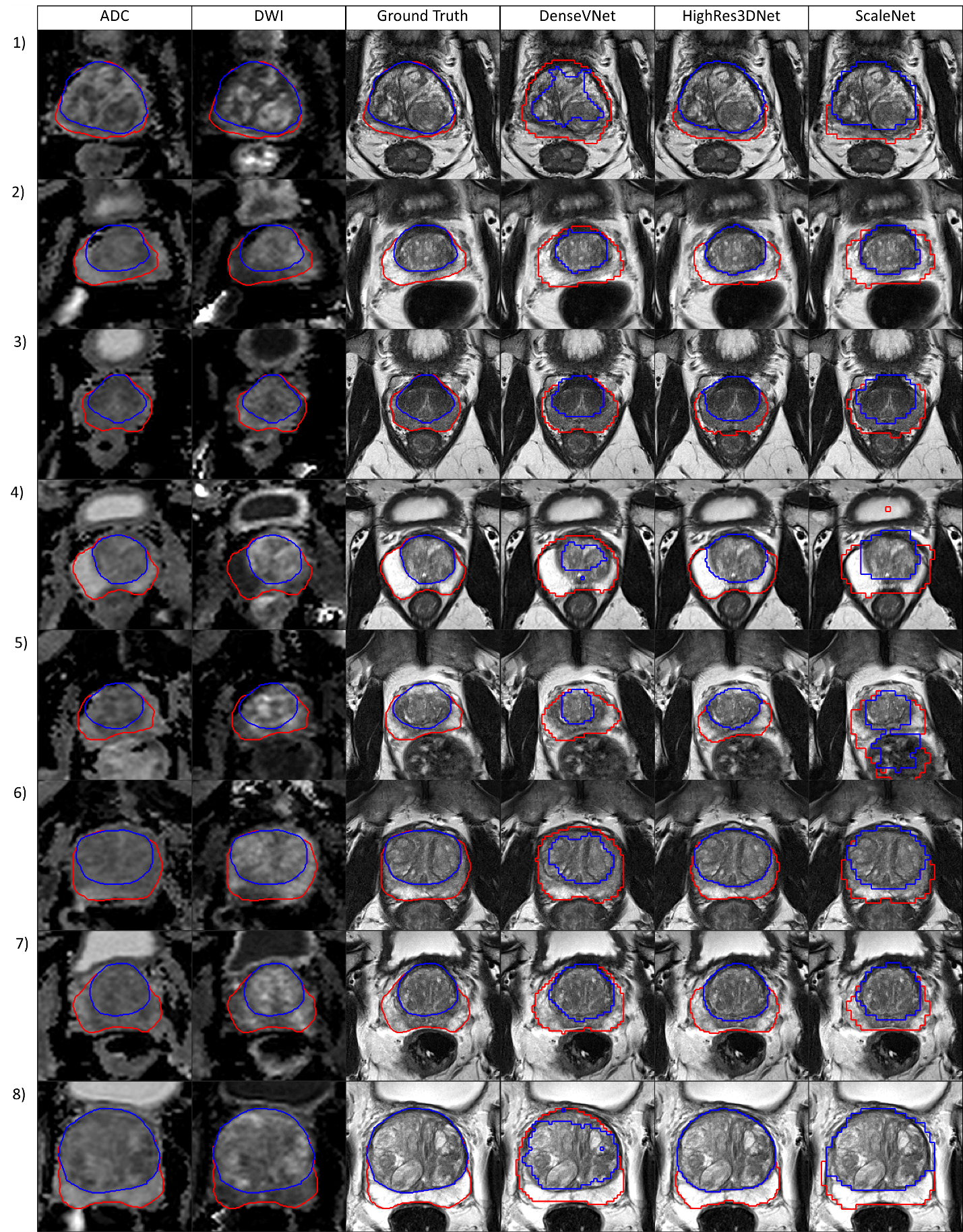


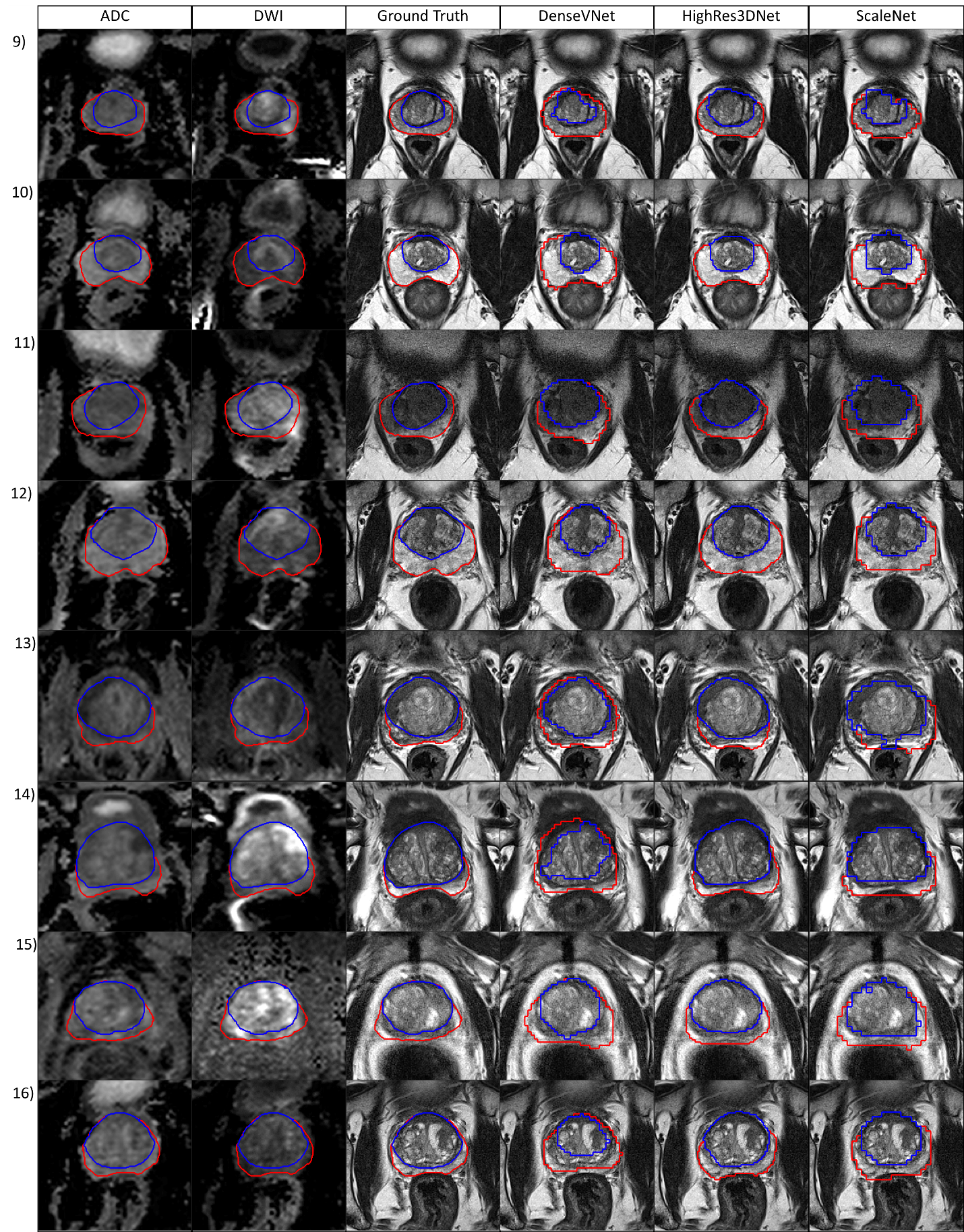

Supplement: Supplementary Materials — Supplementary Table 1: overview of publicly available online prostate-related imaging datasets. Supplementary Figure 1: transverse views of the ADC and DWI images with the respective segmentations of PZ and CG of ground truth, DenseVNet, HighRes3DNet, and ScaleNet for the remaining 16 test subjects. [file 8861035.f1.docx]
